# Trends in Antiparkinsonian Medication Use in New Zealand: 1995–2011

**DOI:** 10.1155/2014/379431

**Published:** 2014-03-04

**Authors:** T. L. Pitcher, M. R. MacAskill, T. J. Anderson

**Affiliations:** ^1^Department of Medicine, University of Otago, Christchurch, P.O. Box 4345, Christchurch 8140, New Zealand; ^2^New Zealand Brain Research Institute, 66 Stewart Street, Christchurch 8011, New Zealand; ^3^Department of Neurology, Canterbury District Health Board, Private Bag 4710, Christchurch 8140, New Zealand

## Abstract

Prescribing trends for medications are influenced by development of new drugs, changes in knowledge about efficacy and side effects, and priorities set by funding agencies. Changes in the utilization of antiparkinsonian agents in the outpatient community in New Zealand were investigated by using the national prescription database for the period 1995–2011. The dispensed volumes of antiparkinsonian agents were converted into number of defined daily doses per 1000 inhabitants per day for analysis. Increases in the dispensed volumes of levodopa (77%), amantadine (350%), and catechol-o-methyl transferase inhibitors (326%) occurred during the study period. Conversely, decreases in the dispensed volumes of anticholinergics (48%), selegiline (82%), and dopamine agonists (6.2%) were observed. New Zealand has seen a substantial increase of the amount of levodopa dispensed in the past 17 years. This increase appears to be related to an increase in the number of people taking the medication. We are unable to extrapolate this change to an increase in the prevalence of PD, given levodopa is used in the treatment of a number of medical conditions. The changes in other antiparkinsonian medications largely reflect changes in availability (increases in entacapone and ropinirole) and best practice treatment (declines in anticholinergics, selegiline, and tolcapone).

## 1. Introduction 

Antiparkinsonian agents are a group of drugs that are primarily used in the treatment of the neurodegenerative disorder, Parkinson's disease (PD). In PD the nigrostriatal dopamine pathway is severely compromised and the antiparkinsonian agents work to counteract the defective dopamine pathway or modulate supporting chemical pathways. To date there are no agents proven to slow the progression of PD. The antiparkinsonian agents are used for symptom relief and people with the disease face continual changes to their medication regimes to maintain optimal relief.

The most common antiparkinsonian agent used for the treatment of PD is levodopa, the precursor to dopamine. Other antiparkinsonian agents include dopamine receptor agonists, catechol-o-methyl transferase inhibitors (COMTIs), monoamine oxidase B inhibitors (MAOIs), anticholinergics, and amantadine. Changes in the rates of utilization of each antiparkinsonian agent over time will reflect changes in the number of people taking the medication, changes in clinical practice, and medication availability. Such changes, however, might not be due solely to their utilization in the treatment of PD. Despite being classified as antiparkinsonian agents, these drugs are also used for the treatment of other conditions. For example, levodopa can be used for the treatment of restless legs syndrome and gait apraxia, anticholinergics are used for the treatment of extrapyramidal side effects of antidopaminergic agents, dopamine agonists are used for the treatment of restless legs syndrome and to reduce prolactin secretion, and amantadine has also been used as an antiviral drug.

Globally the population is ageing. In 2010 the average life expectancy at birth worldwide was 67.5 years and 73.3 years for males and females, respectively. In New Zealand life expectancy was 78.6 years and 82.7 years. This represents a greater than 10-year increase in life expectancy compared to 1970 estimates (males 56.4 years and females 61.2 years, worldwide) [1]. This increase in life expectancy will lead to an increase in the prevalence and duration of treatment for age-related conditions.

New Zealand has a publicly funded healthcare system, so that all eligible residents receive free or subsidised health and disability services. This includes medications, with most day-to-day and many specialised medications being funded. A copayment is generally required with consumers paying NZD 3 (approximately USD 2.33) per dispensed medication during the period 2004 to 2012. This was increased to NZD 5 per dispensed medication in 2013. Since 2004, once an individual or family group reaches 20 prescriptions within a 12-month period, any subsequent prescriptions do not require a copayment, thus limiting the personal annual medication cost to NZD 60 (prior to 2013). Generally, a three-month supply of medicine is provided with each dispensing.

Since 1993, The Pharmaceutical Management Agency (PHARMAC), a government agency, has been responsible for decisions on what drugs will be funded in the public health system, the subsequent bulk purchase of these drugs, and management of drug supply. Decisions on whether a drug will become funded are based on a range of criteria including, clinical benefits and risks, availability and suitability of existing medications, cost effectiveness to the health system as a whole, impact on the pharmaceutical budget, cost to individual consumers, and Ministry of Health priorities. Medical specialist advisory groups (e.g., Neurologist Advisory Panel) exist to provide information to PHARMAC about specialist medications, but these panels have no direct decision making power.

Following the dispensing of a medication, community pharmacies submit reimbursement claims. Information contained within the prescription and details of the subsidy paid are documented within a centrally maintained database, which extends back to 1995. Thus, there is a rich data source available to study community use of medications in New Zealand.

The aim of this study was to investigate the changes in antiparkinsonian agent use in New Zealand during the 17-year period from 1995 to 2011 and to estimate the cost to the national health system of providing these medications.

## 2. Methods

### 2.1. Drug Volumes

Data on the consumption of antiparkinsonian agents in New Zealand were extracted from the national prescription database for the period 1st January 1995 to 31st December 2011. Drugs included in the analyses were those that were indicated for use in PD and funded by the New Zealand government [2]. They corresponded to the Anatomical and Therapeutic Chemical Classification System (ATC; [3] N04 antiparkinson drug category, which includes dopaminergic agents (N04B): levodopa formulations (levodopa with benserazide, levodopa with carbidopa); dopamine agonists (apomorphine, bromocriptine, lisuride, pergolide, and ropinirole); COMTIs (tolcapone and entacapone); MAOIs (selegiline); and amantadine. Anticholinergic agents from the N04A category (procyclidine, orphenadrine, and benztropine) were also included. Data on the MAOI, rasagiline, the dopamine agonist, pramipexole, and the levodopa and entacapone combined formulation, are not included in this analysis as they were not funded for use in New Zealand during the study period.

Volumes of antiparkinsonian agents dispensed in each year were converted using the World Health Organization's defined daily dose metric, which is the *“assumed average maintenance dose per day for a drug used for its main indication in adults”* [3]. The defined daily dose values used were taken from the 2012 edition and are listed in Table 1. National population estimates, issued by Statistics New Zealand at June 30th each year, were used to standardise the volumes of medications, expressed as the number of defined daily doses per 1000 inhabitants per day (DID). New Zealand had an estimated national population of 4.4 million on 30th June 2011 [4].

Medications dispensed within hospitals are not included in this database; however, medications used by residents in rest homes are included; thus the available data is on medication use by the outpatient community. This may lead to an underestimation of the total medication volume consumed. It is likely, however, that volumes used within the hospital setting would be small and therefore have little influence on the overall trends during the study period.

### 2.2. Financial Costs

The financial cost of supplying antiparkinsonian agents in New Zealand was estimated for the 1st July 2010 to 30th June 2011 financial year. During this year PHARMAC spent NZD 706.1 million (approximately USD 550 million) on community pharmaceuticals.

The study was granted ethical approval by The New Zealand Multi-region Ethics Committee.

## 3. Results

### 3.1. Drug Volumes

**Figure 1:**
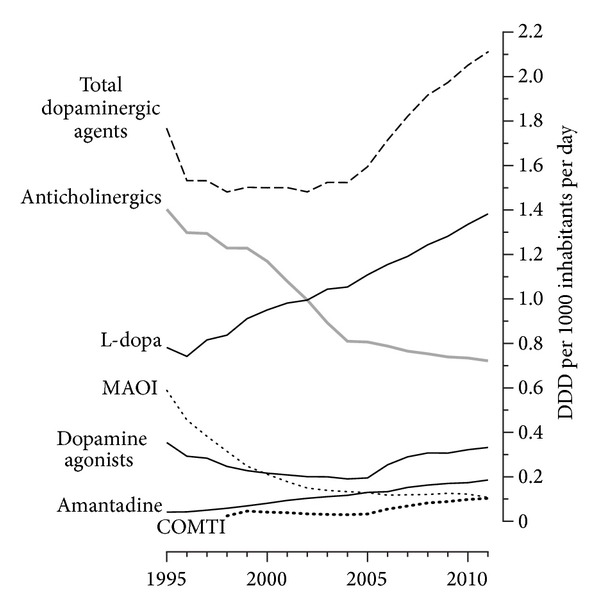
Change in the volume of antiparkinsonian agent groups in New Zealand 1995–2011. Total dopaminergic agents refer to ATC category N04B and exclude anticholinergics. L-dopa: levodopa, MAOI: monoamine oxidase inhibitor, and COMTI: catechol-o-methyl transferase inhibitors. DDD: defined daily dose.

The volume of dopaminergic agents (N04B category) used in New Zealand has increased by 19.6%, with a marked upward trend from 2005 onwards. Full details of DID for all antiparkinsonian agents during the study period are provided in Table 2 and Figure 1 illustrates the change in antiparkinsonian agent groups over the study period.

The volume of levodopa used during the period increased by 77%, with the DID increasing from 0.78 in 1995 to 1.38 in 2011, Figure 2(a). This increase was linear, driven by use of levodopa with carbidopa, whereas use of levodopa with benserazide remained static.

**Figure 2:**
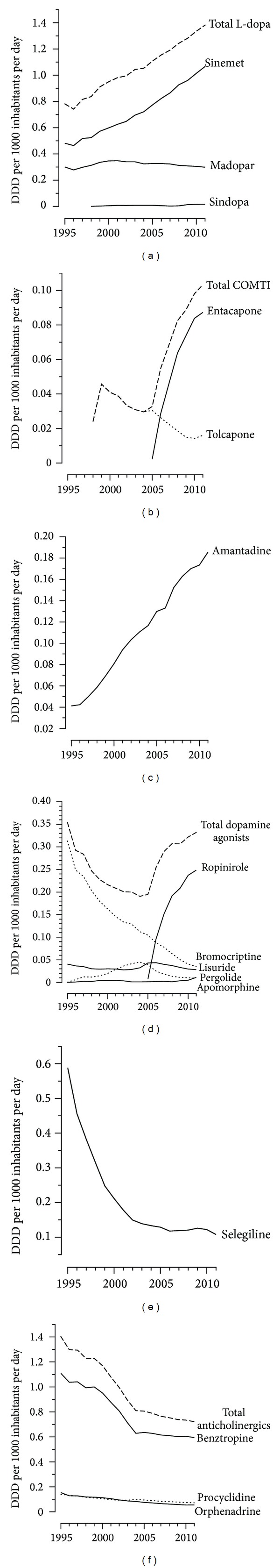
Change in volumes of individual antiparkinsonian agents by drug group. (a) Levodopa (L-dopa), (b) catechol-o-methyl transferase inhibitors (COMTI), (c) Amantadine, (d) dopamine agonists, (e) selegiline, and (f) anticholinergics. DDD defined daily dose.

Other dopaminergic agents to increase were the COMTIs and amantadine. Use of the COMTIs increased markedly (326%) since being made available for use in 1998, Figure 2(b). Since this time, tolcapone volumes have decreased despite retention of funding, while entacapone use has increased substantially since it was made available in 2005. Prescribed amantadine volumes have increased linearly during the study period, with an overall increase of 350%, Figure 2(c).

Dopamine agonist use decreased slightly (6.2%) during the study period. The patterns of change in individual dopamine agonists are shown in Figure 2(d). Since its availability in 2005, ropinirole has shown a substantial increase in use, while bromocriptine use has declined steadily in the past 17 years. There has been a large relative increase in the volume of apomorphine use; pergolide use also increased, while lisuride use declined.

Use of the only MAOI available in New Zealand, selegiline, has decreased substantially (82%) over the study period, sharply in the initial years; however rates of use have plateaued since 2003, Figure 2(e).

New Zealand has experienced a decline (49%) in the use of anticholinergic agents. The magnitude of decline in the three available anticholinergic agents was similar, although the absolute volumes are markedly different. For benztropine, the most prescribed anticholinergic use declined initially, with rates plateauing after 2004. The overall volumes of orphenadrine and procyclidine used were small, with both showing a gradual decline during the study period, Figure 2(f).

### 3.2. Financial Costs

The financial cost of supplying antiparkinsonian agents in New Zealand during the 2010/11 financial year was NZD 5.2 million (approximately USD 4 million). Overall this is a very small (<0.1%) portion of the total community medication spend. The cost of supplying levodopa accounted for 50% of the total antiparkinsonian agent total, COMTIs 18%, dopamine agonists 17.8%, amantadine 8.9%, anticholinergics 4.4%, and selegiline 0.6%.

## 4. Discussion

New Zealand experienced an increase (19%) in the use of dopaminergic agents during the 17-year period 1995 to 2011. One of the main contributors to this increase was a large increase in the volume of levodopa being dispensed. Possible reasons for this increase include (1) an increase in the number of people being treated for PD. Such a connection between levodopa use and PD prevalence is, however, difficult to confirm from this type of data, (2) an increase in the duration of treatment of PD per patient, due to increased lifespan, resulting in higher mean daily doses of levodopa. This is possible given that the population is ageing and people are likely to be living longer with the condition and continuing to experience symptoms requiring levodopa therapy, (3) a change in treatment strategy, such that there may be a trend to treating PD patients with levodopa early, rather than waiting until they are very symptomatic and/or starting dopaminergic therapy with levodopa in preference to dopamine agonists, which tend to have more side effects. This could also account for some of the decline seen in dopamine agonist use, and (4) an increase in the use of levodopa for medical indications other than parkinsonian syndromes—for example, restless legs syndrome, senile gait apraxia, and traumatic brain injury. We suspect, however, that the use of levodopa outside of PD is likely to be small and a minor contribution to the overall volume consumed.

Other antiparkinsonian agents to show increased usage over the review period were entacapone, ropinirole, amantadine, pergolide, and apomorphine. The large increases in entacapone and ropinirole use reflect the relatively quick uptake of these agents once they were funded for use in New Zealand. A proportion of ropinirole use will be accounted for by its application in other conditions such as restless legs syndrome, but the extent of such use in New Zealand is unknown.

The increase observed in pergolide use is perhaps surprising given its association with fibrotic reactions [5, 6]. However, patients with a satisfactory response to pergolide are able to continue using it safely with appropriate monitoring. Apomorphine use increased substantially during the study period as a consequence of increased physician familiarity with efficacy and technical aspects of its subcutaneous delivery, as well as the free provision of the infusion pumps by the marketing company. Overall, the volume of both pergolide and apomorphine used in New Zealand is still very low.

The increase in amantadine mirrors the increase in levodopa use. Amantadine was first proposed for the treatment of PD in 1969 and originally employed as an agent to address the cardinal symptoms [7]. Amantadine nowadays is used more for the relief of levodopa-induced dyskinesias [8]. Given the substantial increase in levodopa use described above it is possible that more PD patients will progress to develop levodopa-induced dyskinesias, which in turn triggers treatment with amantadine.

The remaining antiparkinsonian agents demonstrated diminution in use over the review period. The timing of the decline in selegiline and tolcapone use corresponds to the concerns of the safety of these agents [9–12]. The majority of anticholinergic use is likely to be accounted for by the treatment of extrapyramidal side effects induced by antidopaminergic agents used in psychiatric disorders. The lower incidence of extrapyramidal side effects with the newer atypical neuroleptics [13] and the consequent reduced requirement for anticholinergic therapy (for such side effects) could explain this decline in usage. Use of anticholinergic agents for PD treatment will be relatively minor due to the possible negative impacts on cognition, especially in older patients [14].

The decline in bromocriptine prescribing could be related to a move away from its use once newer dopamine agonists became available. Pergolide was first funded for use in New Zealand in 1995, and many people with PD by now would have been initiated on or switched to pergolide. Likewise cabergoline was also first funded in 1995 (but not included in this study as PD is not an indication for its use in New Zealand). Thus, those with endocrinological indications could have been preferentially given cabergoline over bromocriptine. Lisuride use declined over the review period. This decline might be in relation to the availability of newer dopamine agonists.

**Figure 3:**
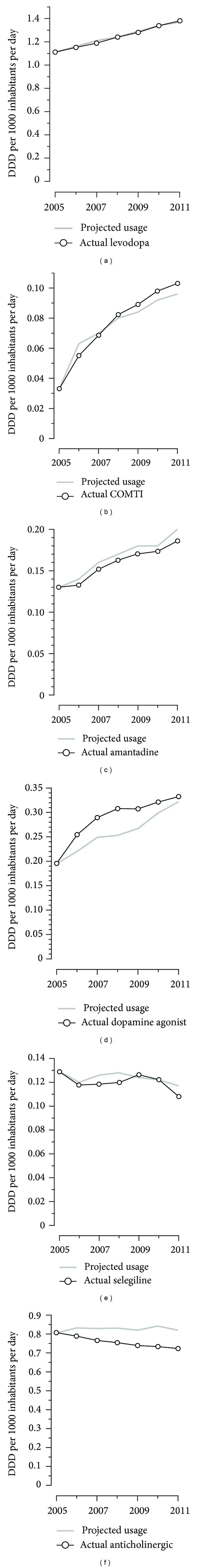
Actual and projected changes in antiparkinsonian agent consumption: 2005–2011. Projected values calculated using the yearly percentage change in the number of people receiving prescriptions for each drug group. (a) Levodopa (L-dopa), (b) catechol-o-methyl transferase inhibitors (COMTI), (c) amantadine, (d) dopamine agonists, (e) selegiline, and (f) anticholinergics.

In an attempt to explain the observed changes in antiparkinsonian agents, we investigated the number of individual people taking medications from each of the antiparkinsonian agent groups on a yearly basis for the period 2005–2011 (the period for which we have individualised data). The expected change in volume of each drug group was estimated by projecting from the 2005 volume, based on the percentage change of number of people receiving prescriptions within each drug group, each year. The expected volume changes are plotted alongside the actual changes in Figure 3. The two lines are relatively close together for most drug groups, with the exception of the dopamine agonists and anticholinergics. This indicates that the changes in volume use are largely driven by changes in the number of people receiving prescriptions. How this might relate to the prevalence of PD is unclear. There have only been two published estimates of PD prevalence in New Zealand, one in 1966 [15] and the other in 1992 [16]. The estimated prevalence rates were 106 and 110 per 100,000 population, respectively. Both these studies were single-center studies, which may result in an underestimation of the national prevalence. There were estimated 630,000 prevalent PD cases in the United States in 2010 [17], which we calculate as a rate of 204 per 100,000 population, twice that of the early New Zealand estimates. It is possible that the number of people with PD in New Zealand has increased since these previous estimates, given that there has been a marked increase (31% during the period 2002 to 2012 [18]) in the proportion of the population in the over sixty-five age group, that is, those most at risk of being diagnosed with the disorder.

The instances where the projected and actual lines deviate away from each other in Figure 3 could perhaps indicate changes in volume that are due to changes in factors other than the number of people taking the medication. In the case of the anticholinergics, the number of people using this drug group has remained fairly constant despite a decline in the volume used. This is consistent with an increase in use of atypical antipsychotics and the reduced extrapyramidal side effects requiring treatment experienced with these drugs. In the case of the dopamine agonists, although the number of people using this class of medication has increased, the expected increase is less than the actual increase. This could indicate that higher doses per person are being prescribed.

Increases in the use of antiparkinsonian agents, including levodopa, have been reported in other countries [19–22]. When comparing the changes in antiparkinsonian agent use in New Zealand to other studies considering similar time periods, we find that the magnitude of increase in levodopa use in New Zealand is similar to that described in a study of the Basque Autonomous Community in Spain [21], but both are somewhat larger than the average increase described across 26 European countries (~15%) [22]. The changes in COMTI agents in New Zealand are similar to changes described in the Basque study. The European study reported decreased use of isolated COMTI, but levodopa with COMTI formulations was included as part of the levodopa total and thus it is difficult to interpret the change in overall COMTI use across Europe. The decline in anticholinergic agent use is similar to that described by Osinaga et al. [21], with European countries also experiencing an average decrease [22].

Changes in the other antiparkinsonian agent groups described here are contrary to the changes described elsewhere, as follows: (1) there was a small decline (6.2%) of dopamine agonist use in New Zealand compared to substantial increases in use elsewhere [21, 22] and (2) substantial decline (82%) in the use of selegiline in New Zealand compared to a modest increase in the Basque study (21.6%) and a small increase in the average volume of MAOI use across Europe. Reasons for these differences could reflect variability in availability of individual drugs or differences in clinical practice. For example, rasagiline, a MAOI, was included in the European study but not in the Basque study or this current study and the dopamine agonist pramipexole was not included in this study, as it was not available during the study period; thus direct comparisons across studies are difficult.

The financial burden of supplying antiparkinsonian agents in New Zealand is small, being less than 0.1% of the total national pharmaceuticals budget. Half of this cost is associated with the provision of levodopa, the mainstay of PD drug treatment. Given the predicted increase in the mean population age in the future we would expect to see the financial cost of supplying these medications to increase, unless there is a significant reduction in manufacturing or supply costs.

There are two main limitations of the study: firstly, the use of a relatively small population (although the nationwide aspect would be considered an advantage) that exists within a highly regulated health system where treatment regimens are established within the confines of a public-funded drug schedule. However, the country does have access to a full range of antiparkinsonian agents that allow for best practice to be followed when treating the predominant user group, patients with PD. Despite a delay in getting access to newer treatment options, for example, rasagiline, pramipexole, and the levodopa and entacapone combined formulation, we suggest that the trends described here for individual drugs will not be that dissimilar to other countries, as evidenced by the comparison to antiparkinsonian agent use in the Basque and European studies [21, 22]. A second limitation is that we are unable to attribute observed changes in drug use to specific medical conditions. This information is not recorded in the prescription database and would require access to individual medical records, which is outside the scope of this study. Having such data would allow for more specific disease-related conclusions to be made.

This study provides a descriptive overview of how the use of antiparkinsonian agents in New Zealand has changed over the past 17 years. It appears that many of the increases in drug volumes consumed are a direct result of increases in the number of people taking the medications. Another potential influence on medication usage is marketing by pharmaceutical companies. New Zealand and the United States are the only two countries to allow direct-to-consumer advertising (NZ since 1981) for prescription medicines. The authors are unaware of any public advertising campaigns relating to antiparkinsonian medications; the lack of such campaigns is likely due to the relatively small market share serviced by these medications. Specific medications maybe advertised in publications of special interest groups, but the effects of such advertisements would be difficult to quantify. Advertising to medical doctors may have some effect on prescribing behaviour, but this would be most evident with medications coming onto the market soon after release by the pharmaceutical companies, something that does not happen with antiparkinsonian medications in New Zealand. With the delay in getting antiparkinsonian medications to market, knowledge of the medication and potential benefits and risks are well established through experiences in other countries, and so advertising is likely to be less effective. Private health insurance in New Zealand is not compulsory and, currently, less than a third of New Zealanders are covered by private health insurance, with the majority of policies only covering nonurgent elective surgical procedures [23]. Thus, access to nonfunded medications by individuals, through insurance, would be rare and not accounted for in this dataset.

It will be pertinent to investigate if the upward trends in use of levodopa, amantadine, entacapone, and ropinirole continue in the future and how such trends might be influenced by the introduction of new medications into the market. For example, the dopamine agonist pramipexole was funded by PHARMAC in November 2012 and will compete with other dopamine agonists for market share in New Zealand. Detailed epidemiological research is required to confirm if the described increase in consumption of PD related medications is associated with an increase in the prevalence of PD in New Zealand.

## Figures and Tables

**Table 1:** Defined daily dose amounts for antiparkinsonian agents available in New Zealand.

Antiparkinsonian agent	Defined daily dose (mg)
Levodopa with decarboxylase inhibitor	600
COMTIs	
Tolcapone	450
Entacapone	1000
Amantadine	200
Dopamine agonists	
Ropinirole	6
Apomorphine	20
Pergolide	3
Lisuride	0.6
Bromocriptine	40
MAOIs	
Selegiline	5
Anticholinergics	
Benztropine	2
Orphenadrine	200
Procyclidine	25

MAOIs: monoamine oxidase inhibitors; COMTIs: catechol-o-methyl transferase inhibitors.

**Table 2:** Defined daily doses per 1000 inhabitants per day of antiparkinsonian medications prescribed to New Zealand outpatients (1995–2011).

	1995	1996	1997	1998	1999	2000	2001	2002	2003	2004	2005	2006	2007	2008	2009	2010	2011	Change (%)
Total dopaminergic agents	**1.76**	**1.53**	**1.53**	**1.48**	**1.50**	**1.50**	**1.50**	**1.48**	**1.52**	**1.52**	**1.59**	**1.71**	**1.82**	**1.92**	**1.97**	**2.05**	**2.11**	**19.6**
L-dopa total	0.78	0.74	0.82	0.84	0.91	0.95	0.98	0.99	1.04	1.05	1.11	1.15	1.19	1.24	1.28	1.34	1.38	76.9
Madopar	0.30	0.28	0.30	0.31	0.34	0.35	0.35	0.34	0.34	0.32	0.33	0.33	0.32	0.31	0.31	0.30	0.30	−0.4
Sinemet	0.48	0.46	0.52	0.52	0.57	0.60	0.63	0.65	0.70	0.72	0.77	0.82	0.87	0.93	0.96	1.01	1.07	121.7
Sindopa				<0.001	<0.01	0.01	0.01	0.01	0.01	0.01	0.01	0.01	<0.01	<0.01	0.01	0.02	0.02	9536.4
Amantadine	0.04	0.04	0.05	0.06	0.07	0.08	0.09	0.10	0.11	0.12	0.13	0.13	0.15	0.16	0.17	0.17	0.19	350.5
COMTIs total				0.02	0.05	0.04	0.04	0.03	0.03	0.03	0.03	0.05	0.07	0.08	0.09	0.10	0.10	326.0
Tolcapone				0.02	0.05	0.04	0.04	0.03	0.03	0.03	0.03	0.03	0.02	0.02	0.01	0.01	0.02	−33.8
Entacapone											<0.01	0.03	0.05	0.06	0.07	0.08	0.09	3759.6
Dopamine agonist total	0.35	0.29	0.28	0.25	0.23	0.22	0.21	0.20	0.20	0.19	0.19	0.25	0.29	0.31	0.31	0.32	0.33	−6.2
Apomorphine	<0.001	<0.001	<0.01	<0.01	<0.01	<0.01	<0.01	<0.01	<0.01	<0.01	<0.01	<0.01	<0.01	<0.01	<0.01	<0.01	0.01	98403.2
Bromocriptine	0.31	0.25	0.23	0.20	0.18	0.16	0.15	0.13	0.13	0.11	0.10	0.09	0.08	0.06	0.05	0.04	0.04	−88.8
Lisuride	0.04	0.04	0.03	0.03	0.03	0.03	0.03	0.03	0.03	0.03	0.04	0.04	0.04	0.04	0.03	0.03	0.03	−30.3
Pergolide	<0.001	0.01	0.01	0.01	0.01	0.02	0.03	0.04	0.04	0.04	0.04	0.03	0.02	0.01	0.01	0.01	0.01	2477.3
Ropinirole											0.01	0.10	0.15	0.19	0.21	0.24	0.25	3349.8
Selegiline	0.59	0.46	0.38	0.31	0.25	0.21	0.18	0.15	0.14	0.13	0.13	0.12	0.12	0.12	0.13	0.12	0.11	−81.7
Anticholinergic total	1.40	1.30	1.29	1.23	1.23	1.17	1.08	0.99	0.89	0.81	0.81	0.79	0.77	0.75	0.74	0.73	0.72	−48.6
Benzotropine	1.11	1.04	1.04	0.99	1.00	0.95	0.88	0.81	0.71	0.63	0.64	0.63	0.62	0.61	0.60	0.60	0.59	−46.4
Orphenadrine	0.15	0.13	0.13	0.12	0.12	0.11	0.11	0.09	0.09	0.08	0.08	0.07	0.07	0.06	0.06	0.05	0.05	−64.4
Procyclidine	0.14	0.13	0.13	0.12	0.11	0.11	0.10	0.09	0.09	0.10	0.09	0.09	0.08	0.08	0.08	0.08	0.07	−48.7

COMTIs: catechol-o-methyl transferase inhibitors; L-dopa: levodopa.
